# Cryptides Identified in Human Apolipoprotein B as New Weapons to Fight Antibiotic Resistance in Cystic Fibrosis Disease

**DOI:** 10.3390/ijms21062049

**Published:** 2020-03-17

**Authors:** Rosa Gaglione, Angela Cesaro, Eliana Dell’Olmo, Rocco Di Girolamo, Luca Tartaglione, Elio Pizzo, Angela Arciello

**Affiliations:** 1 Department of Chemical Sciences, University of Naples Federico II, Via Cintia 21, I-80126 Naples, Italy; rosa.gaglione@unina.it (R.G.); angela.cesaro@unina.it (A.C.); eliana.dellolmo@unina.it (E.D.); rocco.digirolamo@unina.it (R.D.G.); luca.tartaglione.lt@gmail.com (L.T.); 2 Department of Biology, University of Naples Federico II, Via Cintia 21, I-80126 Naples, Italy; elipizzo@unina.it; 3 Istituto Nazionale di Biostrutture e Biosistemi (INBB), I-00136 Rome, Italy

**Keywords:** antibiotic resistance, cystic fibrosis, antimicrobial peptides, host defense peptides, cryptides, anti-biofilm peptides, synergistic effects

## Abstract

Chronic respiratory infections are the main cause of morbidity and mortality in cystic fibrosis (CF) patients, and are characterized by the development of multidrug resistance (MDR) phenotype and biofilm formation, generally recalcitrant to treatment with conventional antibiotics. Hence, novel effective strategies are urgently needed. Antimicrobial peptides represent new promising therapeutic agents. Here, we analyze for the first time the efficacy of three versions of a cryptide identified in human apolipoprotein B (ApoB, residues 887-922) towards bacterial strains clinically isolated from CF patients. Antimicrobial and anti-biofilm properties of ApoB-derived cryptides have been analyzed by broth microdilution assays, crystal violet assays, confocal laser scanning microscopy and scanning electron microscopy. Cell proliferation assays have been performed to test cryptide effects on human host cells. ApoB-derived cryptides have been found to be endowed with significant antimicrobial and anti-biofilm properties towards *Pseudomonas* and *Burkholderia* strains clinically isolated from CF patients. Peptides have been also found to be able to act in combination with the antibiotic ciprofloxacin, and they are harmless when tested on human bronchial epithelial mesothelial cells. These findings open interesting perspectives to cryptide applicability in the treatment of chronic lung infections associated with CF disease.

## 1. Introduction

Cystic fibrosis (CF) is a rare autosomal recessive disease affecting 1 in 2500 newborns in Europe [1]. More than 2000 mutations have been identified in the Cystic Fibrosis Transmembrane conductance Regulator (CFTR) gene and have been associated with the disease. CFTR gene encodes a chloride ion channel whose malfunctioning causes the production of viscous secretions coating the airway epithelia [2,3]. This phenomenon is responsible for the accumulation of trapped microbes, including *Pseudomonas aeruginosa*, with consequent deterioration of lung tissue and impairment of respiratory functions [4]. Indeed, chronic respiratory infections and inflammation are the main causes of death in CF [5]. Despite aggressive antibiotic treatments, *Pseudomonas* strains often grow in CF lungs and lead to chronic and recalcitrant infections characterized by a robust host inflammatory response [6,7]. Pulmonary infections due to the Gram-negative *P. aeruginosa* strain are the main cause of lung decline and death in patients suffering from CF [8,9,10]. *P. aeruginosa* colonization of host tissues is mediated by an initial attachment of bacteria to epithelial cells [11,12], followed by internalization into cells [13,14,15,16]. This phenomenon protects bacteria from host defense mechanisms and from the killing action of conventional antibiotics that hardly enter epithelial cells [17]. This is generally responsible for systemic diffusion of bacteria and for the consequent chronic nature of *P. aeruginosa* lung infections [18]. Moreover, chronic inflammation and mucus provide an environment favorable to the development of resistance phenotype for bacteria in biofilms, thus hampering antibiotic efficacy [19]. *Burkholderia* species also cause serious challenges in CF patients, even if infections associated with these strains are relatively rare [20]. Indeed, a main post-transplant complication is represented by infections caused by multidrug resistant (MDR) bacteria, with the *Burkholderia* species recognized as significant contributors to CF morbidity and mortality associated with increased post-transplant death rate [21,22]. Conventional antibiotics generally appear ineffective and their prolonged use is responsible for the development of the MDR phenotype. The concomitant decrease in the pharmaceutical industry research pipeline for novel antimicrobial agents during the last three decades has, thus, resulted in an urgent need for the discovery of novel effective antimicrobial strategies [23]. In this scenario, naturally occurring antimicrobial peptides (AMPs), or their derivatives, represent an appealing source for the generation of new therapeutic agents able to treat chronic MDR bacterial infections [24,25]. AMPs are produced by all living organisms as the first line of defense against invading microorganisms, and the majority of them are characterized by net positive charge at neutral pH and by the tendency to form amphipathic structures in a hydrophobic environment [26,27]. So far, hundreds of naturally occurring AMPs have been isolated and characterized as highly efficacious, safe, and tolerable antimicrobials [28,29]. Being able to selectively interact with bacterial cytoplasmic membranes in a manner not dependent upon specific receptors, AMPs are generally endowed with broad-spectrum antimicrobial activity [30,31], and several of them have been reported to combat biofilms because of their potent bactericidal activity and their ability to first penetrate and then to disorganize biofilm structures [32]. Furthermore, AMPs frequently synergize with antimicrobial compounds to repress molecular pathways leading to biofilm development [32]. Here, we analyze for the first time the antimicrobial and anti-biofilm properties of two recently characterized AMPs [33] towards *Pseudomonas* and *Burkholderia* strains clinically isolated from CF patients. AMPs under test have been identified in human apolipoprotein B (ApoB) by using a bioinformatic method developed by our research group [33,34,35,36,37,38,39,40,41]. Indeed, it is increasingly evident that eukaryotic proteins, with functions not necessarily related to host defense, act as sources of “cryptic” bioactive peptides released upon proteolytic processing by bacterial and/or host proteases [42,43,44]. We previously characterized two variants of the cryptide identified in human ApoB (residues 887–922), i.e., peptides ApoB887–923 and ApoB887–911 [33]. These two host defense peptides (HDPs), recombinantly produced in bacterial cells, have been here named r(P)ApoB_L_^Pro^ and r(P)ApoB_S_^Pro^ because of the presence of a Pro residue becoming the N-terminus of the peptides released by the acidic cleavage of an Asp-Pro bond [33,36]. Here, we also characterized a further peptide, i.e., a version of the longest peptide characterized by the presence of an Ala residue instead of a Pro residue in position six of peptide sequence, here named r(P)ApoB_L_^Ala^. Peptides r(P)ApoB_L_^Pro^ and r(P)ApoB_S_^Pro^ have been previously found to be endowed with antimicrobial, anti-biofilm, wound healing and immunomodulatory properties, and are able to synergistically act in combination with either conventional antibiotics or EDTA [33]. On the other hand, peptides have been found to be neither toxic nor hemolytic towards mammalian cells [33]. It has been also demonstrated that electrostatic interactions between negatively charged bacterial membranes and positively charged ApoB-derived AMPs play a key role in mediating peptide toxicity, although they are strongly influenced by the composition of negatively charged bacterial surfaces and by defined extracellular microenvironments [35]. Here, we demonstrate that the three ApoB-derived cryptides exert significant antimicrobial and anti-biofilm effects towards *Pseudomonas* and *Burkholderia* strains clinically isolated from CF patients and that they are able to act in combination with the ciprofloxacin antibiotic, widely used to treat chronic lung infections in CF patients [45]. Furthermore, ApoB-derived cryptides have been found to be not toxic when tested on human bronchial epithelial mesothelial cells. Altogether, these findings open interesting perspectives to peptide applicability, suggesting the possibility to develop in the future successful combinatorial therapeutic approaches, based on the concomitant administration of AMPs and conventional antibiotics, with a consequently very low potential to induce a resistance phenotype.

## 2. Results

### 2.1. Evaluation of ApoB-Derived Peptide Effects on Clinically Isolated Baterial Strains

In order to evaluate the ApoB-derived peptide ability to counteract microbial infections in CF, their effects were tested on six clinically isolated bacterial strains, i.e., *P. aeruginosa* RP 73, *P. aeruginosa* KK 27, *P. aeruginosa* 14, *P. aeruginosa* AA2, *Burkholderia multivorans* LMG 17582, and *Burkholderia cenocepacia* LMG 18863. To this purpose, the susceptibility of planktonic bacteria to ApoB-derived peptides was examined by using broth microdilution method [33] that allows the measurement of minimum inhibitory concentration (MIC) values. As reported in Table 1, the three ApoB-derived peptides under test were found to exert antimicrobial effects on three out of six bacterial strains tested. In particular, bacterial strains *P. aeruginosa* RP 73, *P. aeruginosa* KK 27, and *B. multivorans* LMG 17582 were found to be susceptible to ApoB-derived peptide antimicrobial activity, with MIC_100_ values ranging from 5 to 40 μM (Table 1). Peptide r(P)ApoB_L_^Ala^ was found to be the most active in directly killing bacterial cells (Table 1).

### 2.2. Evaluation of ApoB-Derived Peptide Anti-biofilm Activity on Clinically Isolated Baterial Strains

#### 2.2.1. Evaluation of ApoB-Derived Peptide Anti-Biofilm Activity by Microtiter Plate Assay

**Figure 1:**
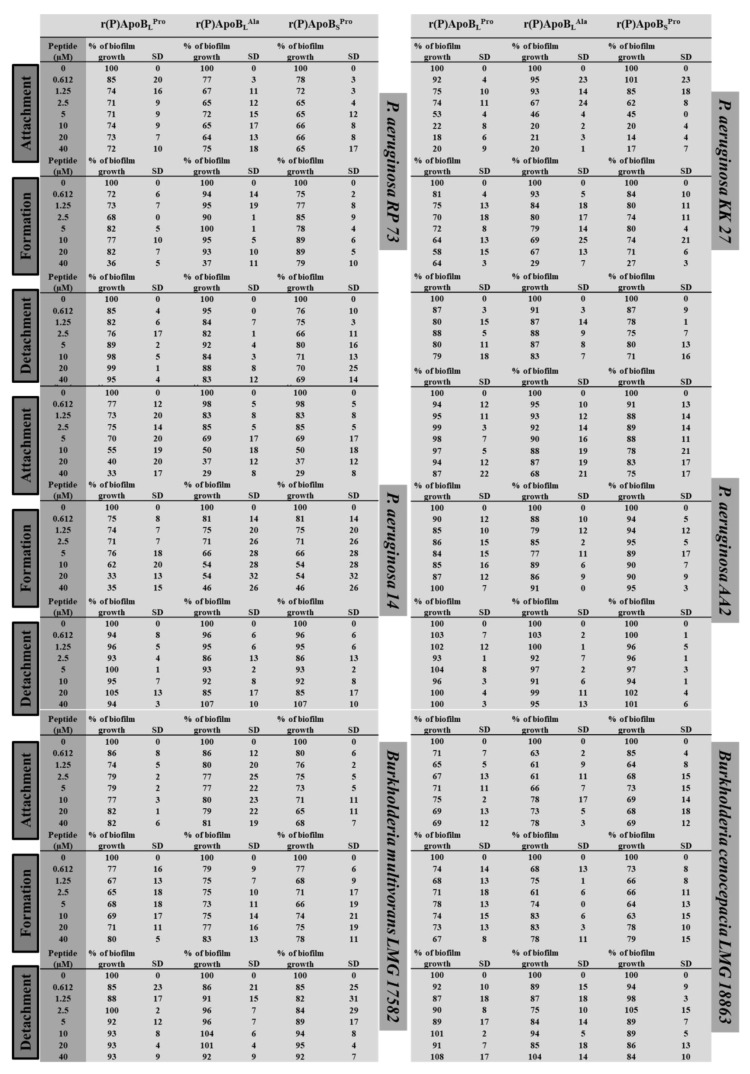
Anti-biofilm activity of r(P)ApoB_L_^Pro^, r(P)ApoB_L_^Ala^, and r(P)ApoB_S_^Pro^ peptides on *P. aeruginosa* RP 73, *P. aeruginosa* KK 27, *P. aeruginosa* 14, *P. aeruginosa* AA2, *B. multivorans* LMG 17582, and *B. cenocepacia* LMG 18863 in MHB medium.

To evaluate whether recombinant ApoB-derived peptides are endowed with anti-biofilm activity, analyses were performed on clinically isolated bacterial strains *P. aeruginosa* RP 73, *P. aeruginosa* KK 27, *P. aeruginosa* 14, *P. aeruginosa* AA2, *B. multivorans* LMG 17582, and *B. cenocepacia* LMG 18863 in 0.5X Mueller Hinton Broth (MHB). By following different experimental approaches, peptide effects were tested on the three main stages of biofilm development, such as attachment, formation and detachment. To test peptide effects on biofilm attachment, following overnight growth, a bacterial culture was diluted into MHB medium containing increasing concentrations of the peptide under test (0–40 μM), and incubated for 4 h at 37 °C [33]. When, instead, peptide effects were tested on biofilm formation, the experimental procedure described above was followed with the only exception that bacterial cells were incubated with increasing concentrations of peptides for 24 h at 37 °C [33]. Finally, the effects of ApoB-derived peptides were tested on biofilm detachment [33]. In each case, following incubation with peptides, biofilm was analyzed by staining with crystal violet. As shown in Figure 1, ApoB-derived peptides have been found to be effective on biofilm attachment, with the greatest effects obtained in the case of *P. aeruginosa* KK 27 and *P. aeruginosa* 14 bacterial strains for all the three peptides under test. In the case of biofilm formation, the greatest effects were found to be exerted by r(P)ApoB_L_^Ala^ and r(P)ApoB_S_^Pro^ on *P. aeruginosa* 14 (~50% inhibition) (Figure 1). Even more interestingly, about 30%–40% biofilm eradication was observed in the case of *B. cenocepacia* LMG 18863 upon treatment with 2.5 μM r(P)ApoB_L_^Ala^ (Figure 1). A similar effect was obtained upon treatment of *P. aeruginosa* KK 27 preformed biofilm with 2.5 µM r(P)ApoB_S_^Pro^ (Figure 1). Moreover, about 20% biofilm eradication was observed upon treatment of *P. aeruginosa* RP 73 with very low concentrations (1.25 μM) of r(P)ApoB_S_^Pro^.

Altogether, obtained data indicate that peptides exert anti-biofilm effects even on bacterial strains not sensitive to their direct antimicrobial activity. In most of the cases, significant anti-biofilm effects were detected at peptide concentrations (1.25–2.5 μM) lower than those required to directly kill planktonic cells (Table 1 and Figure 1). Data reported in Figure 1 represent the mean ± standard deviation (SD) of at least three independent experiments.

#### 2.2.2. Evaluation of ApoB-Derived Peptides Anti-Biofilm Activity by Laser Scanning Confocal Microscopy

**Figure 2:**
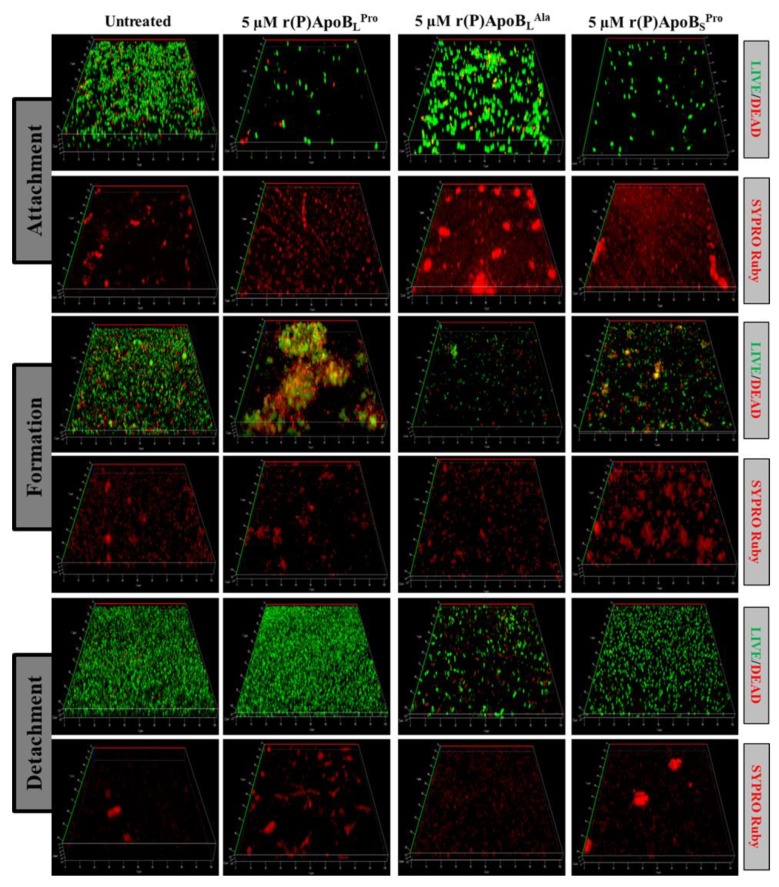
Effects of r(P)ApoB_L_^Pro^, r(P)ApoB_L_^Ala^, and r(P)ApoB_S_^Pro^ peptides on *B. cenocepacia* LMG 18863 biofilm attachment, formation and detachment. Biofilm cells were stained by using LIVE/DEAD BacLight bacterial viability kit (Molecular Probes, Eugene, OR, USA) containing 1:1 ratio of Syto-9 (green fluorescence, all cells) and propidium iodide (PI, red fluorescence, dead cells) and FilmTracer™ SYPRO^®^ Ruby biofilm matrix staining (Invitrogen™, F10318). Images are 3D projections of biofilm structure obtained by laser scanning confocal z-stack using Zen Lite 2.3 software. All images were taken under identical conditions.

**Figure 3:**
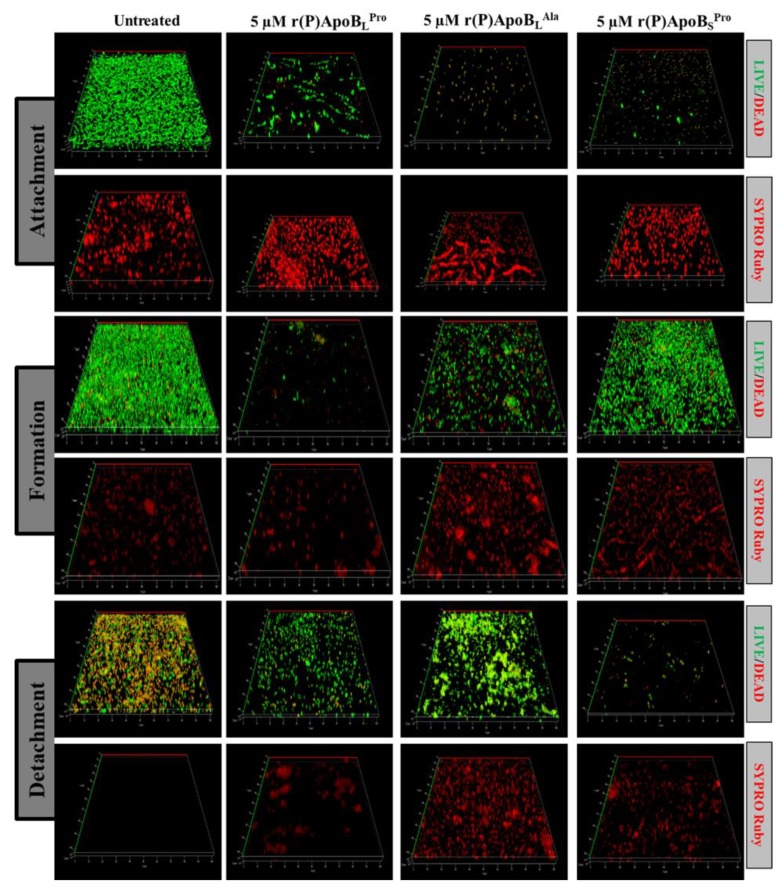
Effects of r(P)ApoB_L_^Pro^, r(P)ApoB_L_^Ala^ and r(P)ApoB_S_^Pro^ peptides on *P. aeruginosa* 14 biofilm attachment, formation and detachment. Biofilm cells were stained by using LIVE/DEAD BacLight bacterial viability kit (Molecular Probes, Eugene, OR, USA) containing 1:1 ratio of Syto-9 (green fluorescence, all cells) and propidium iodide (PI, red fluorescence, dead cells) and FilmTracer™ SYPRO^®^ Ruby biofilm matrix staining (Invitrogen™, F10318). Images are 3D projections of biofilm structure obtained by laser scanning confocal z-stack using Zen Lite 2.3 software. All images were taken under identical conditions.

**Figure 4:**
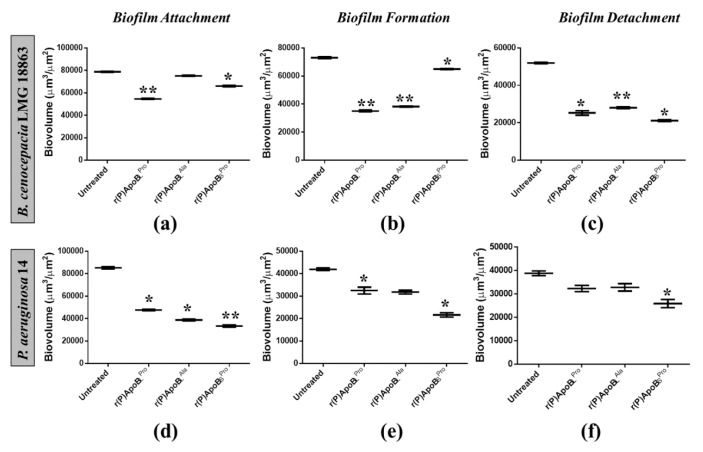
Analysis of the effects of r(P)ApoB_L_^Pro^, r(P)ApoB_L_^Ala^ and r(P)ApoB_S_^Pro^ peptides on biofilm attachment (**a**,**d**), formation (**b**, **e**) and detachment (**c**,**f**) in the case of *B. cenocepacia* LMG 18863 (**a**–**c**) and *P. aeruginosa* 14 (**d**–**f**). Biovolume (µm^3^/µm^2^) was measured by using Zen Lite 2.3 software. Significant differences were indicated as * *p* < 0.05 or ** *p* < 0.01 for treated versus control samples.

In order to further investigate anti-biofilm properties of ApoB-derived peptides, analyses were also performed by confocal laser scanning microscopy (CLSM). For this approach, we selected two bacterial strains not responsive to ApoB-derived peptides direct antimicrobial activity, such as *B. cenocepacia* LMG 18863 and *P. aeruginosa* 14. Peptide effects on biofilm attachment, formation and detachment were evaluated upon sample staining with LIVE/DEAD BacLight bacterial viability kit. Analyses revealed that all three peptides are able to affect biofilm attachment and formation in the case of *B. cenocepacia* LMG 18863 (Figure 2). Even more interestingly, peptides are able to affect pre-formed biofilm with the strongest effects observed in the presence of r(P)ApoB_L_^Ala^ (Figure 2). By staining bacterial biofilm with SYPRO^®^ ruby dye, which is able to specifically stain biofilm extracellular matrix, the appearance of highly fluorescent aggregates is clearly evident upon treatment with peptides (Figure 2), thus indicating that peptides induce strong alterations of biofilm matrix architecture, as previously reported for different anti-biofilm agents [46]. Similar results were obtained also in the case of *P. aeruginosa* 14 (Figure 3). These findings are also supported by biofilm biovolume determinations by CLSM reported in Figure 4, that indicate a strong and significant effect of r(P)ApoB_S_^Pro^ peptide on biofilm eradication in the case of both bacterial strains (Figure 4c,f). Furthermore, peptides have been found to exert significant effects on biofilm biovolume when attachment is tested (Figure 4a,d), except for r(P)ApoB_L_^Ala^ for which no significant reduction in biovolume is observed, although a disaggregating effect is clearly evident (Figure 2 and Figure 3). This might be due to the fact that, upon treatment with r(P)ApoB_L_^Ala^ peptide, planktonic cells escape from biofilm by floating, with a consequent significant contribution to biovolume. Altogether, these findings confirm that peptides are able to exert significant anti-biofilm effects even on bacterial strains not sensitive to their direct antimicrobial activity.

#### 2.2.3. Evaluation of ApoB-Derived Peptides Anti-Biofilm Activity by Scanning Electron Microscopy

**Figure 5:**
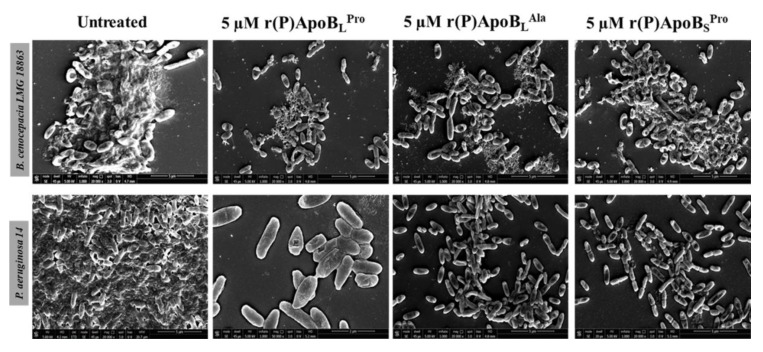
Morphological analyses of *B. cenocepacia* LMG 18863 (top panel) and *P. aeruginosa* 14 (lower panel) preformed biofilms by SEM. Representative images are shown upon treatment of bacterial biofilm with 5 μM r(P)ApoB_L_^Pro^, r(P)ApoB_L_^Ala^, and r(P)ApoB_S_^Pro^. Bars 5 μm.

To analyze morphological modifications of bacterial biofilm upon treatment with peptides, scanning electron microscopy (SEM) analyses were also performed on bacterial strains not responsive to peptide direct antimicrobial activity, such as *B. cenocepacia* LMG 18863 and *P. aeruginosa* 14. In untreated samples, bacteria present smooth and intact surfaces and appear embedded into the extracellular biofilm matrix in the case of both bacterial strains (Figure 5). When bacteria are treated with peptides, instead, a significant decrease or disappearance of biofilm matrix is clearly evident with a concomitant decrease of cell density.

### 2.3. Combinatorial Therapeutic Approach

To verify whether ApoB-derived peptides are able to synergistically act in combination with conventional antibiotics to counteract bacterial infections associated with biofilm in CF, CLSM analyses were performed to evaluate the effects of combinations of r(P)ApoB_L_^Pro^ or r(P)ApoB_L_^Ala^ and ciprofloxacin on preformed biofilm. Analyses were performed on *B. cenocepacia* LMG 18863 bacterial strain, since chronic lung infections associated with this strain strongly contribute to CF morbidity and mortality and are generally recalcitrant to conventional antibiotics [21,22]. Effects of r(P)ApoB_L_^Pro^ or r(P)ApoB_L_^Ala^ peptide in combination with ciprofloxacin were tested on preformed biofilm, in order to better simulate clinical conditions.

**Figure 6:**
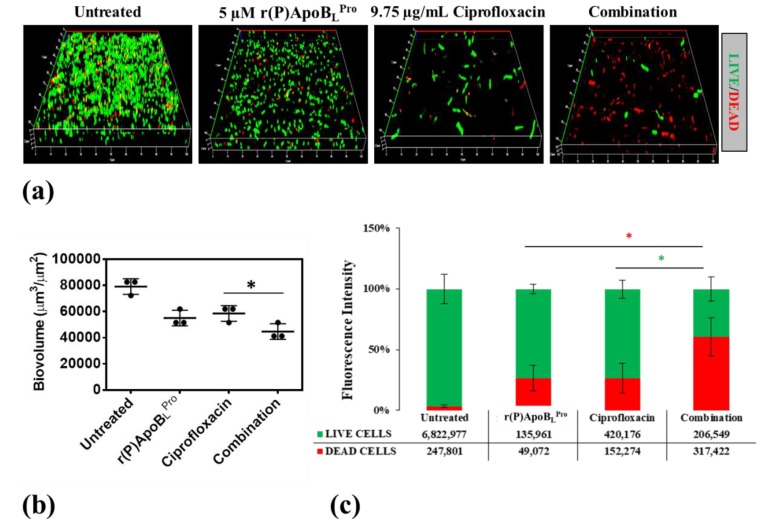
Effects of r(P)ApoB_L_^Pro^, ciprofloxacin and a combination of the two compounds on preformed biofilm (**a**). Biofilm cells were stained by using LIVE/DEAD BacLight bacterial viability kit (Molecular Probes, Eugene, OR) containing 1:1 ratio of Syto-9 (green fluorescence, all cells) and propidium iodide (PI, red fluorescence, dead cells). Images are 3D projections of biofilm structure obtained by laser scanning confocal z-stack using Zen Lite 2.3 software. All images were taken under identical conditions. Biovolume (µm^3^/µm^2^) was measured by using Zen Lite 2.3 software. Significant differences were indicated as * *p* < 0.05 for treated versus control samples (**b**). Numbers of live and dead cells were evaluated by using Zen Lite 2.3 software (**c**).

**Figure 7:**
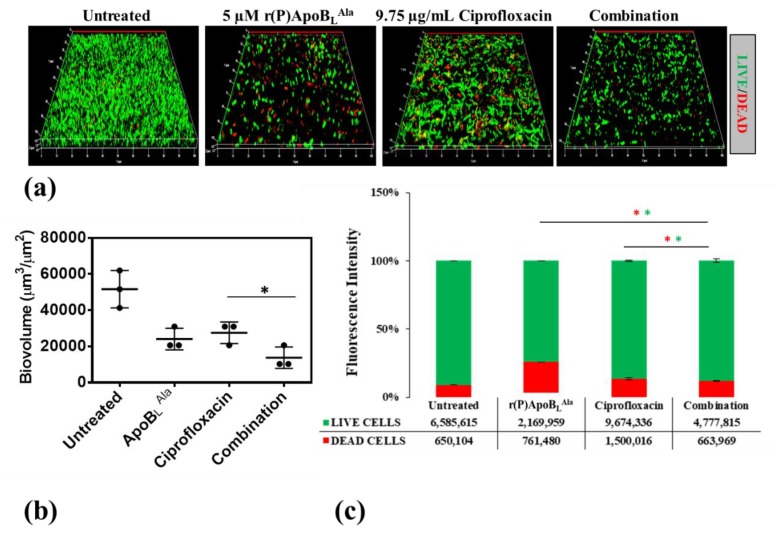
Effects of r(P)ApoB_L_^Ala^, ciprofloxacin and a combination of the two compounds on preformed biofilm (**a**). Biofilm cells were stained by using LIVE/DEAD BacLight bacterial viability kit (Molecular Probes, Eugene, OR) containing 1:1 ratio of Syto-9 (green fluorescence, all cells) and propidium iodide (PI, red fluorescence, dead cells). Images are 3D projections of biofilm structure obtained by laser scanning confocal z-stack using Zen Lite 2.3 software. All images were taken under identical conditions. Biovolume (µm^3^/µm^2^) was measured by using Zen Lite 2.3 software. Significant differences were indicated as * *p* < 0.05 for treated versus control samples (**b**). Numbers of live and dead cells were evaluated by using Zen Lite 2.3 software (**c**).

As shown in Figure 6, by comparing the effects of combinations of r(P)ApoB_L_^Pro^ and ciprofloxacin with the effects of single agents on preformed biofilm, a significantly greater reduction of biofilm biovolume is observed in the case of the sample treated with the compound mixture together with a concomitant increase of the number of dead cells embedded into the biofilm matrix (Figure 6). Similarly, about the effects of combinations of r(P)ApoB_L_^Ala^ and ciprofloxacin on preformed biofilm, a significantly greater reduction of biofilm biovolume is observed in the presence of compounds combination (Figure 7).

### 2.4. Evaluation of Peptide Biocompatibility

**Figure 8:**
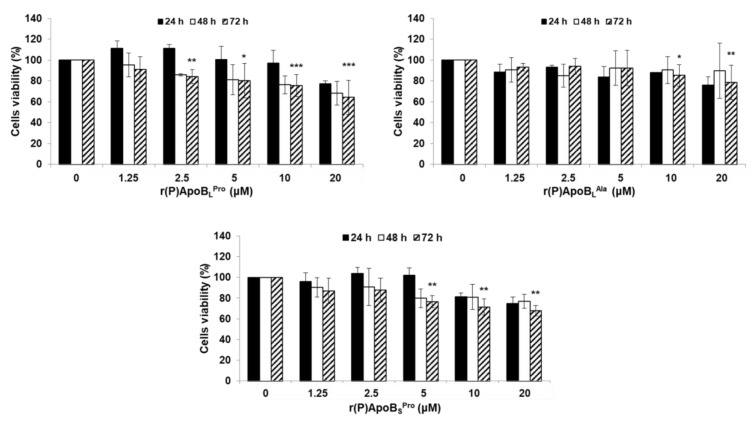
Effects of r(P)ApoB_L_^Pro^, r(P)ApoB_L_^Ala^ and r(P)ApoB_S_^Pro^ peptides on the viability of BEAS cells. Cell viability was assessed by MTT assays, and expressed as the percentage of viable cells with respect to controls (untreated cells). Error bars indicate standard deviations obtained from at least three independent experiments, each one carried out with triplicate determinations. Significant differences were indicated as * *p* < 0.05, ** *p* < 0.01 or *** *p* < 0.001 for treated versus control samples.

Peptide applicability in therapeutic approaches aimed at counteracting bacterial infections associated with CF is strongly dependent on the absence of any toxic effect towards host cells. For this reason, biocompatibility assays were performed to test ApoB-derived peptide effects on immortalized human bronchial epithelial mesothelial (BEAS) cells. As shown in Figure 8, only slight toxic effects were detected and the most biocompatible peptide was found to be r(P)ApoB_L_^Ala^. Indeed, in the presence of this peptide, only slight toxic effects were detected upon 72 h treatment and at the highest peptide concentrations tested (Figure 8).

## 3. Discussion

AMPs represent novel promising effective alternative agents to counteract chronic bacterial infections affecting CF patients. Indeed, as these infections are generally recalcitrant to conventional antibiotics because of the development of the MDR phenotype and of biofilm formation, the development of novel therapeutic strategies is strongly necessary. To this purpose, three versions of a cryptide identified in human ApoB [33,35] have been here tested towards six bacterial strains clinically isolated from CF patients, such as *P. aeruginosa* RP 73, *P. aeruginosa* KK 27, *P. aeruginosa* 14, *P. aeruginosa* AA2, *B. multivorans* LMG 17582, and *B. cenocepacia* LMG 18863. ApoB-derived cryptides have been found to exert direct antimicrobial activity towards three out of six bacterial strains tested. Indeed, ApoB-derived AMPs have been found to be active on *P. aeruginosa* RP 73, *P. aeruginosa* KK 27, and *B. multivorans* LMG 17582, with MIC_100_ values ranging from 5 to 40 μM. This is in agreement with recent findings indicating that ApoB-derived cryptides direct antimicrobial activity, although mediated by electrostatic interactions between cationic peptides and negatively charged bacterial membranes, is strongly influenced by chemical composition of LPS molecules exposed on the surface of different strains of *P. aeruginosa* [35]. Indeed, although several bacterial resistance components against antimicrobial peptides have been reported [47], LPS chemical composition has been proposed to play a key role in the case of ApoB-derived cryptide antimicrobial activity [35]. It has been reported that different *Burkholderia* strains present different LPS chemotypes, such as rough, partial rough, or smooth [48]. In particular, in the case of *B. cenocepacia* LMG 18863, the LPS chemotype has been identified as smooth [48]. These differences in LPS chemotype might be responsible for the different susceptibility of *Burkholderia* strains to ApoB-derived cryptides’ direct antimicrobial activity. It also has to be highlighted that several *B. cenocepacia* strains have been reported to be naturally resistant to different classes of antibiotics and even to several antimicrobial peptides [49]. This is probably due to the ability of *B. cenocepacia* strains to acquire a resistance phenotype by modifying the LPS chemical composition by substituting a phosphate group with a cationic charged residue of 4-amino-4-deoxy-L-arabinose (L-Ara4N), with a consequent reduction in membrane negative potential, that plays a key role in the interaction between bacterial membranes and antimicrobial peptides [50,51]. Based on these observations, ApoB-derived cryptides direct antimicrobial activity towards *B. multivorans* LMG 17582 appears to be really interesting. It also has to be considered that, in the case of chronic infections affecting CF patients, the mucus phenotype favors bacterial biofilm formation [20]. Bacteria embedded into biofilm matrix are more resistant to conventional antibiotics for several reasons: i) low antibiotic diffusion rate inside biofilm matrix; ii) bacteria metabolic changes due to nutrients missing, with a consequently lower susceptibility to antibiotics; and iii) appearance of persisted cells recalcitrant to conventional antibiotics and playing a key role in long-term infections [52]. Anti-biofilm cationic amphipathic peptides represent an alternative promising approach to treat infections associated with biofilm formation, since peptides act on biofilm specific targets, such as matrix components and/or highly conserved regulatory mechanisms [53]. Here, we tested the ability of r(P)ApoB_L_^Pro^, r(P)ApoB_L_^Ala^ and r(P)ApoB_S_^Pro^ to affect the biofilm of bacterial strains clinically isolated from CF patients. In particular, we analyzed the ability of ApoB-derived cryptides to interfere with the three main stages of biofilm development, i.e., attachment, formation and detachment [33]. We found that all the three ApoB-derived cryptides are able to exert significant effects on biofilm attachment and formation. Even more interestingly, ApoB-derived cryptides have been found to exert significant anti-biofilm effects even on bacterial strains not sensitive to their direct antimicrobial activity. In particular, ApoB-derived AMPs have been found to affect *P.* *aeruginosa* 14 biofilm attachment and formation and *B. cenocepacia* LMG 18863 preformed biofilm at a very low concentration (2.5 μM). As reported for different AMPs, obtained data allow us to exclude any correlation between peptide direct antimicrobial activity and their anti-biofilm properties. Indeed, peptide IDR-1018 has been reported to be endowed with strong anti-biofilm activity towards a pool of *P.* *aeruginosa* and *Burkholderia* strains in the absence of any direct antimicrobial effect [54]. To deeply characterize ApoB-derived cryptides anti-biofilm activity, we also performed analyses by confocal laser scanning microscopy (CLSM) and scanning electron microscopy (SEM) on *P.* *aeruginosa 14* and *B. cenocepacia* LMG 18863, two strains not sensitive to ApoB-derived AMP’s direct antimicrobial activity. CLSM analyses revealed the ability of ApoB-derived cryptides to alter biofilm architecture, as indicated by the appearance of highly fluorescent aggregates only in treated samples upon staining with SYPRO^®^ Ruby, a dye able to specifically label biofilm extracellular matrix. Accordingly, a significant reduction in biofilm biovolume has been evaluated in the case of samples treated with ApoB-derived cryptides. Furthermore, scanning electron microscopy analyses clearly indicate the ability of ApoB-derived cryptides to disrupt the biofilm matrix of bacterial strains not responsive to the peptides’ direct antimicrobial activity. These observations are in perfect agreement with data reported for peptide 6K-F17, which is able to strongly affect *P. aeruginosa* biofilm by disrupting the extracellular matrix, thus determining a significant decrease of biofilm biovolume [55]. However, CLSM and SEM analyses indicate only a slight increase of bacterial cells death upon treatment with 6K-F17 [55]. Based on obtained results, we also evaluated the possibility to set up effective combinatorial therapeutic approaches by concomitantly administrating ApoB-derived cryptides and conventional antibiotics to bacterial cells. To this purpose, we analyzed the anti-biofilm properties of combinations of r(P)ApoB_L_^Pro^ or r(P)ApoB_L_^Ala^ and the antibiotic ciprofloxacin, which is widely used to treat bacterial infections in CF patients [45]. The effects of compound mixtures have been tested on *P. aeruginosa 14* or *B. cenocepacia* LMG 18863 preformed biofilm. Effects of combinations of r(P)ApoB_L_^Pro^ or r(P)ApoB_L_^Ala^ and ciprofloxacin on preformed biofilm have been found to be stronger than those of single agents, with more severe effects on biofilm biovolume. It has been previously reported that, upon biofilm treatment with the ciprofloxacin antibiotic, a deep alteration of the matrix structure and a strong decrease of biofilm biovolume are immediately observed, probably associated with a high killing rate of bacterial cells embedded into the biofilm matrix [56]. However, upon a prolonged exposure to ciprofloxacin, the activation of specific mechanisms leading to a variation of biofilm phenotype makes the antibiotic ineffective [56]. This phenomenon might be overcome by the development of successful combinatorial therapeutic approaches, which present several advantages over conventional therapeutic treatments based on the administration of single agents. Indeed, several anti-biofilm peptides have been reported to be able to act in synergism with a broad range of conventional antibiotics [57]. This allows us to significantly reduce the effective dose of antibiotics up to 64-fold, with a consequent lower possibility to induce MDR phenotype and to simultaneously reduce effective peptide concentrations [57]. In the case of CAMA peptides, synergism with the conventional antibiotics tobramycin, ciprofloxacin and colistin has been demonstrated in the treatment of *P. aeruginosa* biofilm, with the consequent possibility of reducing antibiotics doses up to 8-fold and peptide concentrations up to 10-fold [58]. Since one of the bottlenecks for the development of successful peptide-based therapies is peptide cytotoxicity, we also tested ApoB-derived cryptides effects on immortalized human bronchial epithelial mesothelial (BEAS) cells, and found that peptides are biocompatible, since slight toxic effects are detected only upon 72 h cell treatment and at the highest peptide concentrations tested. Altogether, obtained findings open interesting perspectives to the applicability of ApoB-derived cryptides in the treatment of bacterial chronic infections associated with biofilm formation and characterized by MDR phenotype, such as those affecting CF patients, and to the development in the future of successful combinatorial therapeutic approaches based on the concomitant administration of peptides and conventional antibiotics.

## 4. Materials and Methods

### 4.1. Materials

All the reagents were purchase from Sigma-Aldrich (Milan, Italy), unless differently specified.

### 4.2. Recombinant Production of ApoB-Derived Peptides

Expression and isolation of recombinant ApoB-derived peptides was carried out as previously described [33,35]. Pro → Ala substitution in position six of the longest peptide was obtained by QuikChange II site-directed mutagenesis performed by using the following primers: primer forward 5’-CATTTTACCCGCTTTCAGCGCAACATGCGGGTG-3’ and primer reverse 5’-GATCCGCATGTTGCGCTGAAAGCGGGTAAACTG-3’.

### 4.3. Bacterial Strains and Growth Conditions

Bacterial strains *P. aeruginosa* RP 73, *P. aeruginosa* KK 27, *P. aeruginosa* 14, *P. aeruginosa* AA2, *B. multivorans* LMG 17582, and *B. cenocepacia* LMG 18863 were kindly provided by Dr. Alessandra Bragonzi (Infection and CF Unit, San Raffaele Scientific Institute, Milan, Italy). Bacterial strains were grown in MHB (Becton Dickinson Difco, Franklin Lakes, NJ, USA) and on Tryptic Soy Agar (TSA; Oxoid Ltd., Hampshire, UK). In all the experiments, bacteria were inoculated and grown overnight in MHB at 37 °C.

### 4.4. Eukaryotic Cells and Growth Conditions

Immortalized human bronchial epithelial mesothelial cells (BEAS) were cultured in high-glucose Dulbecco’s modified Eagle’s medium (DMEM) supplemented with 10% fetal bovine serum and 1% penicillin-streptomycin at 37 °C in the presence of 5% carbon dioxide (CO_2_).

### 4.5. Cell Viability Assays

Peptide effects on eukaryotic cell viability was evaluated by seeding cells in 96-well plates (100 μL/well) at a density of 3×10^3^ cells/well. Upon 24 h, cells were incubated with increasing peptide concentrations (0–40 μM), for 24, 48 and 72 h. At the end of the treatment, cell viability was assessed by the 3-(4,5-dimethylthiazol-2-yl)-2,5-diphenyltetrazolium bromide (MTT) assay. MTT reagent, dissolved in DMEM without phenol red, was added to the cells (100 μL/well) at a final concentration of 0.5 mg/mL. After 4 h at 37 °C, the culture medium was removed and the resulting formazan salts were dissolved by the addition of isopropanol containing 0.1 N HCl (100 μL/well) [41]. Absorbance values of blue formazan were determined at 570 nm by using an automatic plate reader (Synergy™ H4 Hybrid Microplate Reader, BioTek Instruments, Inc., Winooski, VT, USA). Cell survival was expressed as the percentage of viable cells in the presence of the peptide under test, with respect to control cells grown in the absence of the peptide.

### 4.6. Antimicrobial Activity Assays

To test the antimicrobial activity of ApoB-derived peptides, a previously described experimental procedure was used [33]. MIC_100_ values correspond to the lowest concentration of peptide associated with no detectable bacterial growth.

### 4.7. Anti-Biofilm Activity by Crystal Violet Assay

ApoB-derived peptides effects on biofilm attachment, formation and detachment were evaluated as previously described [33]. Optical densities at 595 nm of biofilm stained biomasses were measured by using a microtiter plate reader (Synergy™ H4 Hybrid Microplate Reader, BioTek Instruments, Inc., Winooski, VT, USA).

### 4.8. Anti-Biofilm Activity by CLSM Analyses

Bacterial biofilm was grown on glass cover slips in 24-well plates in 0.5X MHB in static conditions. In particular, bacterial cells from an overnight culture were diluted to about 1 × 10^8^ CFU/mL and then seeded into wells for 4 or 24 h at 37 °C in the presence of the peptide under test, in order to evaluate biofilm attachment and formation, respectively. When effects on preformed biofilm were evaluated, bacterial biofilms were formed for 24 h at 37 °C, and then treated with peptides under test for further 24 h to evaluate their ability to eradicate preformed biofilm. Afterwards, non-adherent bacteria were removed by gently washing samples with sterile phosphate buffer and viability of cells embedded into biofilm structure was determined by sample staining with LIVE/DEAD^®^ BacLight™ Bacterial Viability kit (Molecular Probes, Thermo Fisher Scientific, Waltham, MA, USA), while FilmTracer™ SYPRO^®^ Ruby biofilm matrix dye has been used to stain matrices of biofilms (Invitrogen, Carlsbad, CA, USA). Staining was performed accordingly to manufacturer instructions. Biofilm images were captured by using a confocal laser scanning microscopy (Zeiss LSM 710, Zeiss, Germany) and a 63X objective oil immersion system. Biofilm architecture was analyzed by using the Zen Lite 2.3 software package (Zeiss, Germany). Each experiment was performed in triplicate. All images were taken under identical conditions.

### 4.9. Anti-Biofilm Activity by Scanning Electron Microscopy

To perform scanning electron microscopy (SEM) analyses, *B. cenocepacia* LMG 18863 and *P. aeruginosa 14* cells were incubated with 5 μM r(P)ApoB_L_^Pro^, r(P)ApoB_L_^Ala^ or r(P)ApoB_S_^Pro^ peptides for 24 h at 37 °C. Following incubation, bacterial biofilms were fixed in 2.5% glutaraldehyde. Following overnight incubation, bacterial biofilms were washed three times in distilled water and then dehydrated with a graded ethanol series: 25% ethanol (1 × 10 min); 50% ethanol (1 × 10 min); 75% ethanol (1 × 10 min); 95% ethanol (1 × 10 min); 100% anhydrous ethanol (3 × 30 min). Bacterial biofilms deposited onto glass substrate were sputter coated with a thin layer of Au-Pd (Sputter Coater Denton Vacuum DeskV) to allow subsequent morphological characterization using a FEI Nova NanoSEM 450 at an accelerating voltage of 5 kV with Everhart Thornley Detector (ETD) and Through Lens Detector (TLD) at high magnification.

### 4.10. Statistical Analysis

Statistical analysis was performed using a Student’s t-test. Significant differences were indicated as * *p*  <  0.05, ** *p*  <  0.01 or *** *p*  <  0.001.

## Figures and Tables

**Table 1:** Minimum inhibitory concentration (MIC) values determined for r(P)ApoB_L_^Pro^, r(P)ApoB_L_^Ala^ and r(P)ApoB_S_^Pro^ tested on clinically isolated bacterial strains.

	MIC_100_ (μM)		
	r(P)ApoB_L_^Pro^	r(P)ApoB_L_^Ala^	r(P)ApoB_S_^Pro^
*P. aeruginosa RP* 73	10–20	5–10	20–40
*P. aeruginosa* 14	>40	>40	>40
*P. aeruginosa* AA2	>40	>40	>40
*P. aeruginosa* KK 27	20–40	10–20	20–40
*Burkholderia cenocepacia* LMG 18863	>40	>40	>40
*Burkholderia multivorans* LMG 17582	10–20	10–20	20–40

## Abbreviations

CF	Cystic Fibrosis
MDR	Multidrug Resistance
ApoB	Apolipoprotein B
HDPs	Host Defense Peptides
CFTR	Cystic fibrosis transmembrane conductance regulator
AMPs	Antimicrobial peptides
MIC	Minimum inhibitory concentration
MHB	Mueller Hinton Broth
CLSM	Confocal laser scanning microscopy
SEM	Scanning electron microscopy
PI	Propidium iodide
LPS	Lipopolysaccharide
